# Enhancing Product Performance via a Modified Double-Diaphragm Forming (mDDF) Preform Method for Prepreg Compression Molding of Fiber-Reinforced Polymer Composites

**DOI:** 10.3390/polym17111489

**Published:** 2025-05-27

**Authors:** Shin Kim, Honchung Shin, Kilsung Lee, Sungkyu Ha

**Affiliations:** 1 Department of Mechanical Engineering, Hanyang University, 222 Wangsimri-ro, Seonjong, Seoul 04763, Republic of Korea; kimsh6905@hanyang.ac.kr; 2 Korea Carbon Industry Promotion Agency, 110-11, Ballyong-ro, Deokjin-gu, Jeonju-si 54853, Republic of Korea; chung5505@kcarbon.or.kr; 3 Human Composites Co., Ltd., 152, Jayumuyeok-ro, Gunsan-si 54002, Republic of Korea; ks2994@humancomposites.com

**Keywords:** Prepreg Compression Molding (PCM), thermoset composites, wrinkle elimination, prepreg preforming, Modified Double-Diaphragm Forming (mDDF), draping simulation, forming defect analysis

## Abstract

An enhanced process for shaping thermoset fiber-reinforced composites using Modified Double-Diaphragm Forming (mDDF) in Prepreg Compression Molding (PCM) is proposed to address limitations in conventional forming quality. To minimize surface defects, prepregs were pre-cut to reduce wrinkle formation and trimmed after preforming. Complex geometries were managed through draping analysis, which enabled identification and mitigation of wrinkle-prone regions. A challenging layup configuration (±45/0/90/0/90/0/±45) was selected, and temperature-dependent behavior of the prepreg—such as resin fluidity and wrinkle characteristics—was evaluated from room temperature to 80 °C. Material characterization included tensile, bias extension, bending, friction, and density tests. Forming simulations using AniForm Suite 3.0 incorporated fitted material parameters for predictive analysis. Experimental validation confirmed that the mDDF process significantly improved fiber alignment and eliminated wrinkle defects, especially in 16 previously identified critical zones. The final parts exhibited superior surface quality and dimensional accuracy compared to conventional PCM, highlighting the potential of mDDF for precision manufacturing of complex thermoset composite structures.

## 1. Introduction

As industries move toward lightweighting and improved fuel efficiency, polymer matrix composites are increasingly replacing metals in structural applications. Carbon fiber-reinforced polymer (CFRP) composites have become essential materials in high-performance environments due to their excellent specific strength, stiffness, corrosion resistance, and design flexibility. These materials are typically composed of high-strength fibers—such as carbon or glass—embedded in a polymer matrix, either thermoset or thermoplastic. In particular, thermoset polymer composites have been widely adopted in the aerospace, automotive, and sports equipment industries owing to their high mechanical strength and stiffness [1], thermal stability, chemical resistance, lightweight and design flexibility.

The growing demand for mass production of composite parts has accelerated the adoption of efficient manufacturing processes for polymer composites. Among these, Prepreg Compression Molding (PCM) stands out for its simplicity, cost-effectiveness, and compatibility with thermoset prepregs. PCM involves stacking sheets of pre-impregnated fibers (prepregs) into a mold, then pressing and curing them under heat to form a finished part. This technique is particularly attractive for medium-to-high volume manufacturing of structural parts with moderate complexity. However, as the demand for more geometrically complex and customized parts increases—such as in electric vehicles, drones, and sporting goods—conventional PCM [2] faces performance and quality limitations in their complex shapes.

One major challenge in PCM is the formation of wrinkles and defects during molding, particularly when the prepregs are cut and stacked to fit complex geometries. To stack the curved surface to the prepreg, the prepreg must be cut. Cut of curved prepreg surfaces can degrade fiber alignment, reduce strength, and increase material waste. Additionally, wrinkles formed during compression compromise surface finish and mechanical integrity. These issues are especially critical in high-precision polymer composite components where structural reliability is essential. Hence, there is a growing need to improve PCM-based forming methods to accommodate complex shapes while preserving fiber orientation and minimizing defects.

A promising approach to address these limitations is the integration of preforming techniques into the PCM workflow. Preforming involves reshaping the prepreg laminate into a near-net shape before final molding, allowing for better conformity to mold surfaces and wrinkle control. Furthermore, simulation-driven draping analysis and mechanical property characterization of prepregs can support the optimization of fiber orientations, stacking sequences, and forming parameters. These strategies are particularly effective when applied to thermoset epoxy-based polymer composites, where flow behavior and cure kinetics must be precisely managed.

In this study, we propose a Modified Double-Diaphragm Forming (mDDF) process [3] as an enhancement to conventional PCM, designed to improve shape conformance and mechanical performance with cutting the prepreg but without damage to the product. The mDDF method introduces a semi-curing preforming [4] stage supported by simulation-based wrinkle prediction and experimental characterization of prepreg properties. This approach achieves complex geometries with improved fiber orientation and reduced defects. The paper presents a detailed investigation of the mDDF method, including draping analysis, material testing, and molding trials using thermoset carbon epoxy prepregs, and demonstrates its effectiveness in producing high-quality fiber-reinforced polymer components.

## 2. Manufacturing Process: Conventional PCM and Proposed mDDF Method

Fiber-reinforced polymer (FRP) composites are widely used in industries requiring high strength-to-weight ratios, including automotive, aerospace, and defense sectors. One of the most established forming techniques for these materials is Prepreg Compression Molding (PCM), particularly suitable for thermoset polymer matrices such as epoxy. The PCM process enables repeatable production of complex-shaped composite parts using pre-impregnated (prepreg) fiber layers and thermal curing.

### 2.1. Conventional Prepreg Compression Molding Process

**Figure 1:**
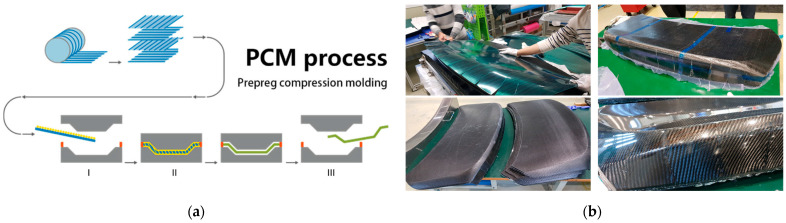
The basic PCM process and connected issues: (**a**) the PCM process (https://www.zjmdc.com/PCM-Mould.html (accessed on 20 May 2025)) and (**b**) issues in the cutting zone.

The PCM process begins with cutting prepreg sheets—typically carbon fiber fabrics impregnated with a thermoset epoxy polymer resin—into the desired shapes based on the mold geometry. These sheets are stacked according to a predetermined lay-up sequence and placed into a heated mold. Compression is applied to consolidate the layers and initiate curing of the polymer matrix. After curing, the part is demolded and trimmed to its final shape. This process is illustrated schematically in Figure 1a.

While PCM is widely adopted for forming flat or moderately contoured parts, several challenges arise when complex geometries are required. These include:

- The need to cut prepreg sheets to conform to curved mold surfaces, which may introduce discontinuities in fiber reinforcement and complicate lay-up procedures.
- Wrinkle formation due to in-plane shear and out-of-plane deformation during compression.
- Fiber misalignment or slippage, especially in multilayered [5] laminates with varied stacking sequences.
- Material waste generated from excessive trimming and off-cuts.

Figure 1b shows an example of the issues commonly encountered in cut and stacked laminates, particularly in zones with double curvature. These challenges can affect both the manufacturing efficiency and the final mechanical properties of the polymer composite parts. To overcome the limitations of the conventional Prepreg Compression Molding (PCM) process, the Modified Double-Diaphragm Forming (mDDF) method was introduced in this study. Specifically, mDDF aims to address key issues such as (1) the reduction in strength and stiffness caused by cutting of prepregs, (2) the challenge of forming without inducing material damage, and (3) the need to maintain stable fiber alignment throughout the forming of complex geometries. The proposed process incorporates a semi-curing preforming step and diaphragm-assisted pressure control, offering a more reliable and defect-minimizing alternative to traditional PCM.

### 2.2. Proposed Modified Double-Diaphragm Forming (mDDF) Method

To address the above limitations of conventional PCM, we propose a Modified Double-Diaphragm Forming (mDDF) method as a process extension. The mDDF method incorporates a preforming [6] stage prior to PCM, intended to improve geometric conformity and without damage to the product.

**Figure 2:**
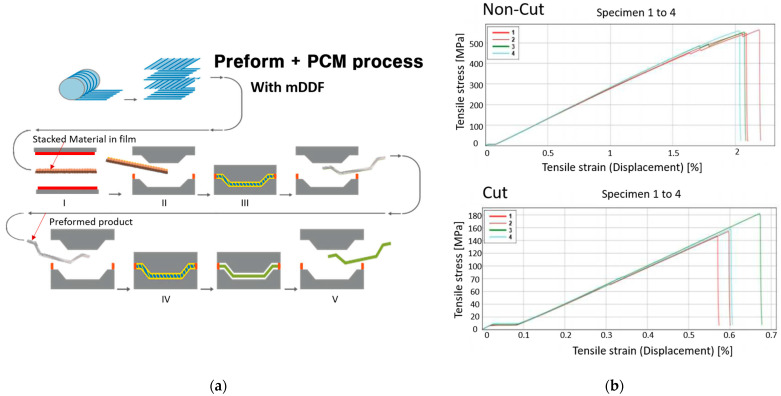
The mDDF process with prepreg preforming and differences in cutting: (**a**) the mDDF process with prepreg preforming and (**b**) CFRP S-S curve.

**Figure 3:**
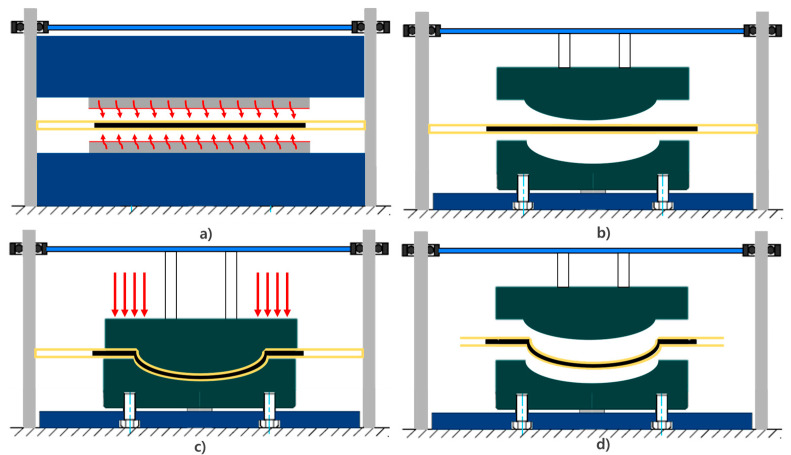
mDDF prepreg preforming machine and process: (**a**,**c**) preform machine and (**b**,**d**) schematic of the Modified Double-Diaphragm Forming process.

The mDDF process proceeds as follows (schematically illustrated in Figure 2 and Figure 3):

- Layup with prepreg: Carbon/epoxy polymer prepreg sheets with unidirectional (UD) and twill weaving are stacked on a flat surface in the same targeted stacking order as (±45/0/90/0/90/0/±45). Do not cut on the product line, only the outside of the product line. This is to prevent crease from entering from the blank holder. Trim this part after preforming.
- Vacuum Bagging with Diaphragms: The laminate is sandwiched between high-stretchable diaphragm films (e.g., Gore-Tex) and enclosed in a vacuum bag to apply uniform pressure.
- Semi-Curing (B-Stage): The bagged laminate is heated to an intermediate temperature, between 47–53% [7] of its glass transition temperature (Tg), allowing the epoxy polymer matrix to partially cure. At this stage, the laminate becomes sufficiently formable while maintaining structural cohesion.
- Forming Step: The semi-cured prepreg stack is rapidly transferred to a forming mold and compressed using a press [8]. This step shapes the laminate into the desired 3D geometry without requiring cuts in the fiber layers.
- Final PCM Molding: The preformed part is subsequently placed into the final PCM mold, where full curing of the polymer matrix is completed under controlled heat and pressure conditions.
- Demolding and Minimal Trimming: The final part is removed from the mold and trimmed as necessary. Due to the preform’s geometric conformance, less post-processing is expected.

The mDDF approach is designed to integrate with existing PCM infrastructure while introducing a preforming mechanism that may help to mitigate issues such as wrinkling, fiber misalignment, and material waste—commonly observed in traditional PCM [9]. The semi-cured state is critical to maintain the formability of the polymer matrix during shaping, and the diaphragm ensures more uniform pressure distribution over complex surfaces.

Further evaluation is required to assess the effectiveness of this method. The following sections describe the material characterization and simulation-based analysis conducted to support the implementation of mDDF.

## 3. Material Characterization for Draping Simulation of the mDDF Process

The accurate simulation of composite laminate deformation during preforming requires a detailed understanding of the mechanical behavior of the prepreg material, particularly in the temperature range relevant to the forming process. In the proposed Modified Double-Diaphragm Forming (mDDF) method, prepregs are processed at a semi-cured (B-stage) state, in which the polymer matrix exhibits viscoelastic characteristics and the laminate remains formable but dimensionally stable [3]. To enable reliable modeling in AniForm Suite 3.0, a complete material card was constructed based on experimental characterization of uncured carbon/epoxy prepregs provided by Hankuk Carbon (SKYFlex system).

Four categories of mechanical tests were performed: tensile testing for elastic stiffness, bias extension testing for in-plane shear behavior, friction testing for interfacial interactions, and bending tests for ply flexibility. All tests were conducted between room temperature and 80 °C to replicate mDDF processing conditions. The resulting data were used as direct input to construct a temperature-dependent, ply-level forming model.

**Figure 4:**
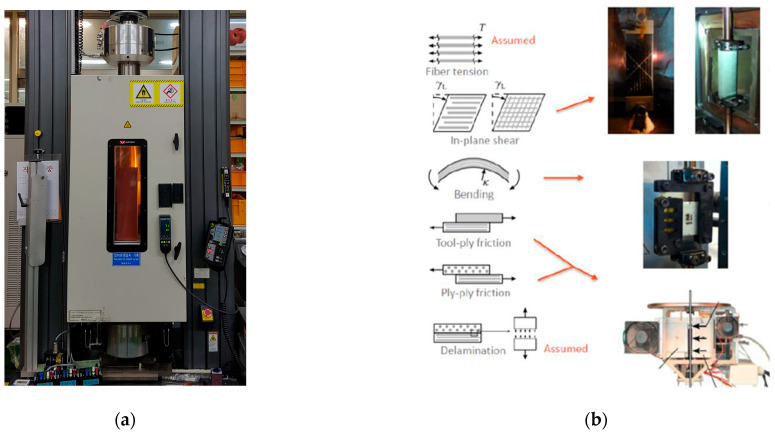
The mechanical properties of prepreg materials subjected to preforming: (**a**) UTM equipment with a climatic chamber and (**b**) the mechanical properties.

For the prepreg draping analysis of the preforming, the mechanical properties of twill and UD materials under varying temperatures were obtained through a tensile test (ASTM D 3039 standard), bias extension test, friction test (ASTM D 1894 standard), and bending test (ASTM D 1388 standard), as shown in Figure 4a. Figure 4b shows the tensile specimen mounted on the tensile test machine using 250kN UTM equipment (Instron) with a climatic chamber, FP-2260 Friction/Peel Tester(Thwing-Albert Instrument company), and fabric stiffness model tester TF113 (TESTEX). The specimen was used in the prepreg state without curing, and the state change was measured under temperature conditions.

### 3.1. Tensile Testing

**Figure 5:**
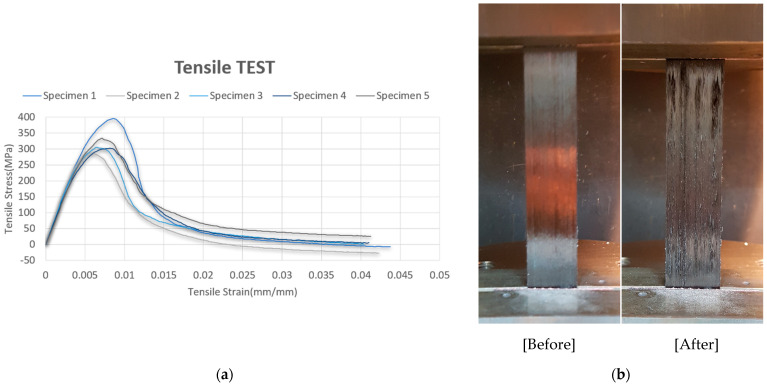
The tensile test of prepreg properties: (**a**) stress–strain curve and (**b**) tensile test before (**left**) and after (**right**).

Uniaxial tensile tests were carried out following ASTM D3039 to determine the axial stiffness of the uncured prepreg in the fiber direction (0°). Five specimens with dimensions of 240 mm × 25 mm × 0.27 mm were tested at 70 °C using a 250 kN Instron universal testing machine equipped with a climatic chamber. The measured stress–strain curves were used to evaluate the fiber-dominated modulus of elasticity. The average elastic modulus across all specimens was 65.5 GPa. Figure 5a presents the typical stress–strain response, and Figure 5b shows the specimen appearance before and after testing.

This tensile modulus was used as the primary input for modeling longitudinal stiffness in unidirectional layers within the forming simulation.

### 3.2. Bias Extension Testing

**Figure 6:**
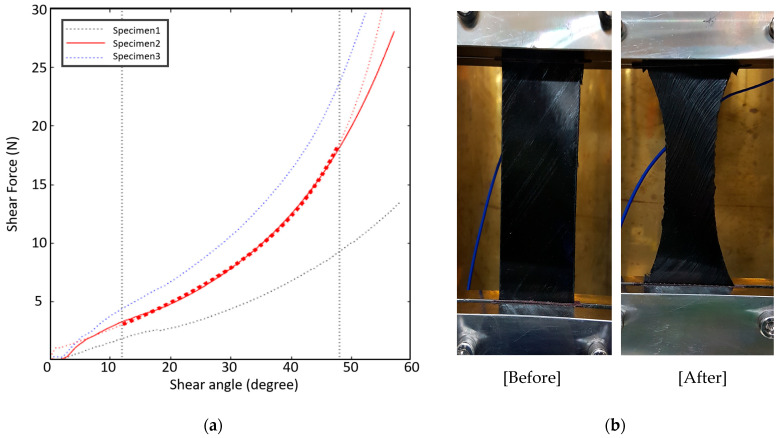
The bias extension test of prepreg properties: (**a**) shear force–shear angle curve and (**b**) bias extension test before (**left**) and after (**right**).

In-plane shear behavior was characterized using bias extension tests on ±45° oriented specimens, with dimensions of 240 mm × 50 mm × 0.27 mm. Testing was performed at 70 °C, and the response was modeled using the *Kelvin–Voigt viscoelastic model*:

$$\sigma \left(t\right)=Ε\varepsilon \left(t\right)+\eta \frac{d\varepsilon (t)}{dt}$$

where E is the elastic modulus and η\etaη is the viscosity. These parameters were optimized using AniForm’s fitting module, based on the experimental shear force–shear angle data. Table 1 summarizes the best-fit parameters, and Figure 6a shows the modeled curve alongside experimental results. Approximately 1600 data points per test were collected to ensure convergence.

The shear modulus and viscosity were essential to simulate [10] inter-ply distortion and wrinkle formation in curved geometries.

### 3.3. Friction Test

Frictional behavior between prepreg layers and between prepreg and tooling surfaces significantly influences slippage and layer shifting during forming. Friction tests were conducted on specimens of 200 mm × 40 mm × 0.125 mm in two configurations: tool–prepreg and prepreg–prepreg contact. The FP-2260 Friction/Peel Tester was used to apply controlled normal loads and slip velocities at 70 °C.

**Figure 7:**
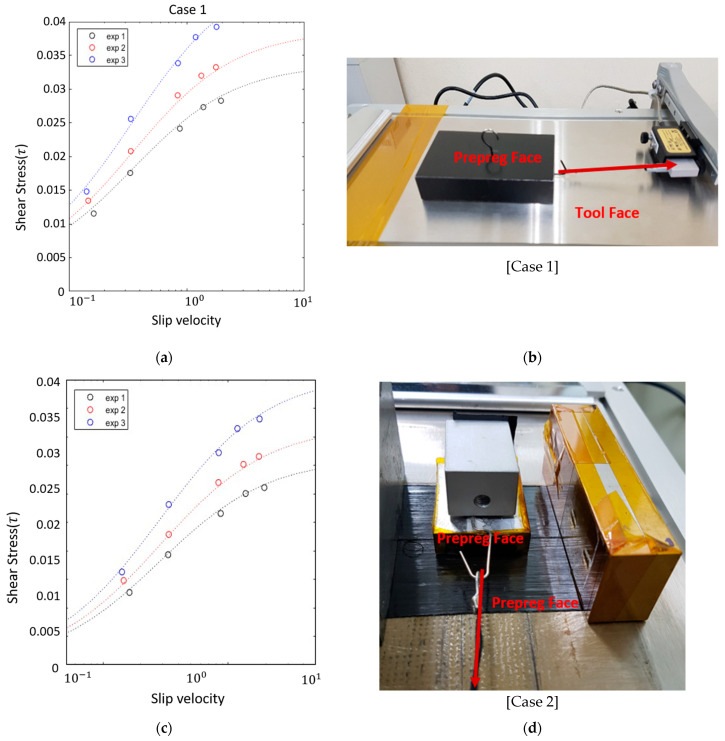
The friction test of prepreg properties: (**a**) shear stress–slip velocity curve of case 1; (**b**) the friction test of the tool face–prepreg face; (**c**) shear stress–slip velocity curve of case 2; (**d**) the friction test of the prepreg face–prepreg face.

The measured traction force was fitted to *a cross-viscosity friction model*:

$$\tau =C\cdot \nu^{n}$$

where τ\tauτ is the shear stress, v is the slip velocity, and C and n are empirical fitting constants. Table 2 provides the fitting results, and Figure 7 illustrates the test configurations and model performance.

### 3.4. Bending Test

**Figure 8:**
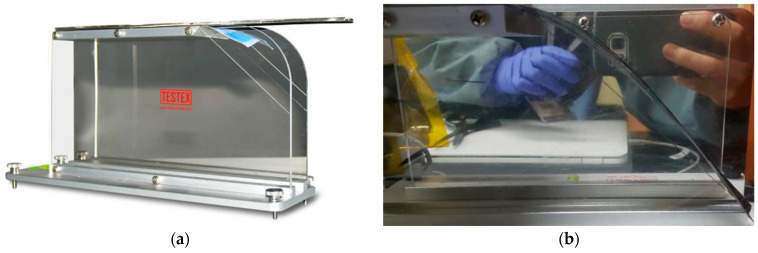
The bending test of prepreg properties: (**a**) the hot plate for the high-temperature test and (**b**) measurement of the overhang length of the fiber at a bending angle of 41.5°.

Flexural stiffness of the prepreg was assessed using the horizontal cantilever method (ASTM D1388). Specimens were 200 mm × 25 mm × 0.27 mm and tested at 70 °C. The overhang length at a bending angle of 41.5° was measured, and bending stiffness D was calculated using the following equation:

$$D=\frac{W\cdot L^{3}}{3\cdot \theta }$$

where W is a real weight, L is overhang length, and θ\thetaθ is the bending angle in radians. Table 3 reports the test results, and Figure 8 shows the measurement setup.

This parameter was crucial in modeling the laminate’s ability to drape over tooling surfaces without generating wrinkles or folds.

## 4. Draping Simulation and Forming Results

**Figure 9:**
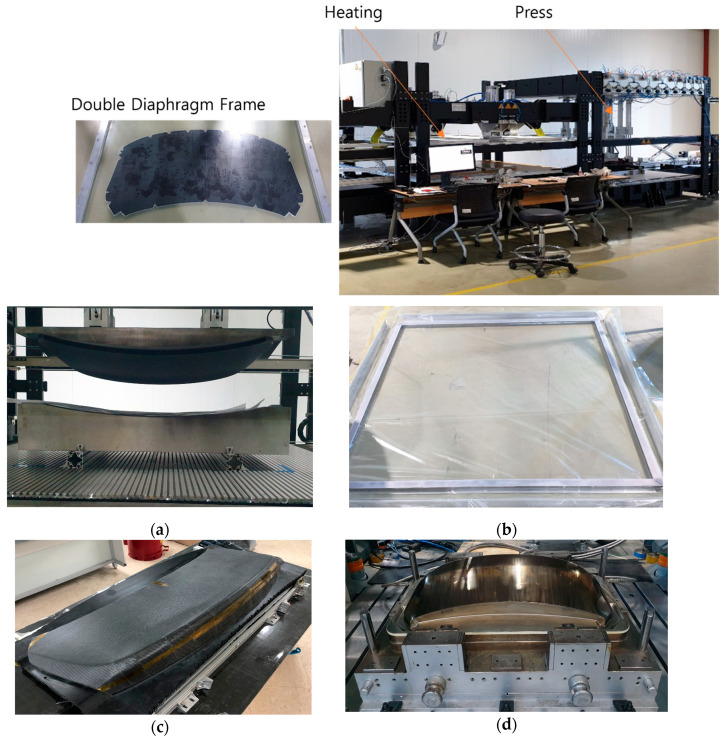
The preform mold and mDDF frame: (**a**) preform mold; (**b**) the Modified Double-Diaphragm Forming frame; (**c**) preformed trimming mold (3D printed); (**d**) PCM mold.

To validate the proposed Modified Double-Diaphragm Forming (mDDF) method for fiber-reinforced polymer composites, draping simulations [11] and preforming trials were performed [12]. The preform machine used in the experiment is from the company CKONI and is shown in Figure 3a. the Modified Double-Diaphragm Forming method was applied to shape and utilize the frame, is shown in Figure 9. Draping simulation provides critical insight into the deformation [13] behavior of the semi-cured polymer prepregs during forming [14], allowing identification of wrinkle-prone regions, fiber misalignment, and thickness variation before experimental molding. Based on the simulation outcomes, preforming strategies such as local cutting and stress relief methods were optimized. This section presents the simulation setup, draping analysis results, and the outcomes of preforming and final molding trials.

### 4.1. Draping Simulation Setup for mDDF Process

To support the implementation of the Modified Double-Diaphragm Forming (mDDF) method, draping simulations were conducted to predict the deformation behavior of the semi-cured thermoset prepregs during the preforming stage. The simulation was designed to capture key factors that influence forming quality [15], including fiber distortion, wrinkling, thickness variation, and ply slippage. Accurate modeling of these deformation phenomena was essential for identifying process limitations and optimizing mold design and lay-up configurations prior to experimental trials.

The simulation environment was developed using AniForm Suite 3.0, a specialized finite element tool for composite forming analysis. A ply-based draping model was generated based on the material card, which incorporated the experimentally measured mechanical properties described in Section 3. These included tensile modulus from uniaxial testing, in-plane shear properties from bias extension tests, interfacial friction data for both ply–ply and tool–ply interactions, and bending stiffness derived from cantilever tests. All material data were calibrated at a forming temperature range of 70 °C, representative of the B-stage cure state of the epoxy matrix.

**Figure 10:**
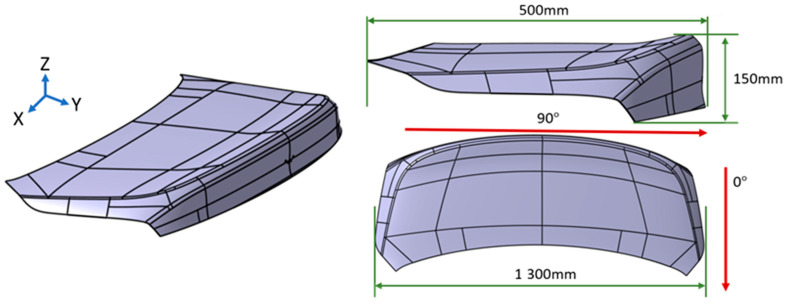
Tool geometry for the case study component. Red lines indicate the direction of the stacking pattern.

The laminate was modeled with a stacking sequence of (±45/0/90/0/90/0/±45), incorporating both unidirectional (UD) and twill weave plies. The thickness of the lay-up corresponded to the target 1.2 mm final part requirement as designed above in Figure 10. A matched tool geometry, consistent with the mold used in the experimental trials, was imported into the simulation platform. The mold featured regions of double curvature and corner transitions, which were expected to challenge fiber alignment and promote wrinkle formation [16].

The simulation workflow included sequential lay-up of the plies, application of gravity and forming pressure, and contact interactions between the laminate and the tooling surfaces. Forming pressure and cycle time were matched to those used in the experimental process to maximize relevance. Boundary conditions were applied to replicate the effect of diaphragm-assisted compaction during vacuum forming, and numerical stabilization was introduced to ensure convergence during large-deformation events.

The output data from the simulation included ply-wise displacement fields, inter-ply shear, thickness variation, fiber angle deviation, and predicted wrinkle zones. These outputs were used not only to assess the feasibility of forming the chosen geometry under semi-cured conditions but also to identify critical regions for process refinement, such as where cuts or localized process modifications might be necessary. The simulation results are discussed in detail in the following section.

**Figure 11:**
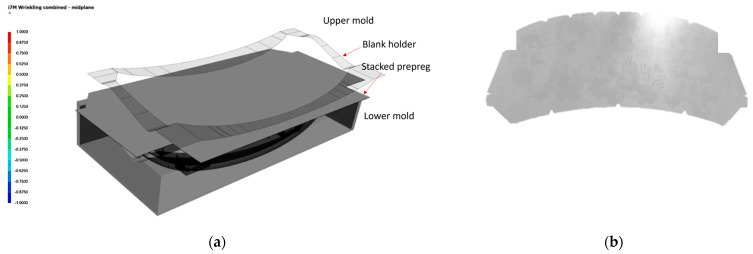
Draping simulation setup: (**a**) draping setup for simulation and (**b**) pre-cut stacked prepreg designed to reduce wrinkling.

**Figure 12:**
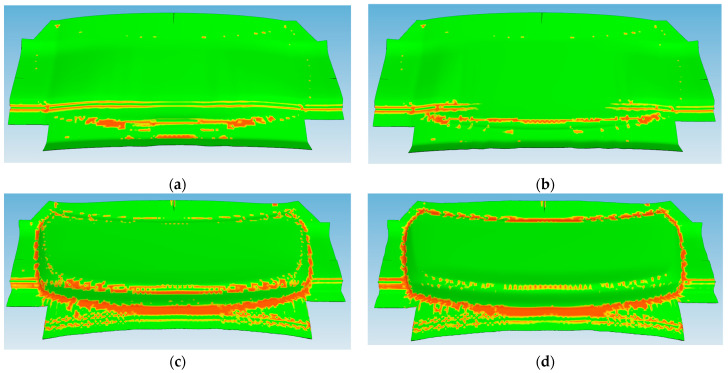
Draping simulation change results: (**a**) step1; (**b**) step2; (**c**) step3; (**d**) step4.

As shown in Figure 11a, draping simulation is set up as Lower mold, stacked prepreg, and upper mold, and diaphragm film is implemented as a bank holder. In Figure 12a, draping analysis shows that wrinkles are concentrated in regions of double curvature and corner transitions. To mitigate these wrinkles, a pre-cut material design was implemented, as shown in Figure 11b.

### 4.2. Draping Simulation Results

The results of the draping simulation provided valuable insight into the deformation behavior of the semi-cured thermoset polymer composite laminate during the mDDF process. The simulation revealed that wrinkle formation was concentrated in specific regions of the laminate, particularly near areas of double curvature and localized thickness transitions. These predicted wrinkle zones were visualized as areas of fiber buckling and excessive local compression, as shown in Figure 12. These results were critical in identifying the need for process adjustments in the form of local stress-relief strategies.

**Figure 13:**
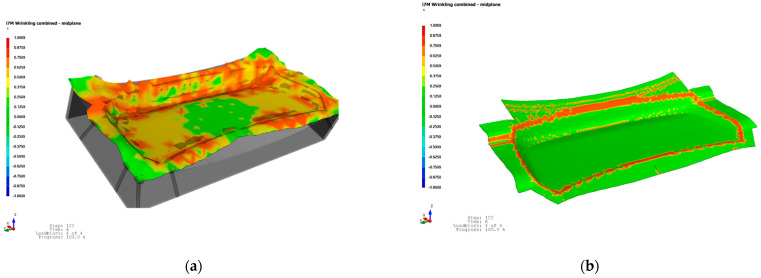
Draping analysis: (**a**) draping analysis status and (**b**) draping analysis result.

To address the wrinkling tendencies observed in the simulation, small, strategically placed cuts were introduced into the laminate during the preforming stage. The draping analysis results in Figure 13a show that wrinkles are concentrated in regions of double curvature and corner transitions. The intent of the cuts was to allow localized ply displacement during forming while minimizing global fiber misalignment. A pre-cut material design was applied to optimize wrinkle reduction, as shown in Figure 11b. This approach led to an optimal outcome with significantly reduced wrinkling, as demonstrated in Figure 13b. The application of these design changes based on simulation feedback helped improve shape conformity and reduced out-of-plane defects [17].

In addition to wrinkle prediction, the simulation highlighted moderate fiber reorientation within the outer plies, especially near corner radii and contoured sections of the mold. However, the core layers of the laminate largely retained their intended orientations, indicating acceptable deformation behavior under the simulated forming conditions. Thickness distribution across the laminate showed minor variations, which were attributed to localized ply shifting and compaction but were within acceptable tolerance for post-molding performance.

Overall, the simulation demonstrated that the proposed material model accurately captured key deformation phenomena such as wrinkling, slippage, and thickness variation. The simulation results were used to inform forming parameters [18] and guide the layout of the prepreg laminate during the experimental preforming process [19] described in the next section.

### 4.3. Experimental Preforming and Final Molding

The Modified Double-Diaphragm Forming (mDDF) process was experimentally implemented using the semi-cured carbon/epoxy prepregs under conditions matched to the simulation setup [20]. The vacuum bagged laminate was placed between high-elongation diaphragm films and subjected to press forming at the B-stage temperature, resulting in a preform that conformed closely to the mold geometry.

The process parameters were as follows: temperature of 70 degrees, temperature of the material (70 degrees), within 5 seconds until pressing, holding time of 10 seconds (Table 4).

**Figure 14:**
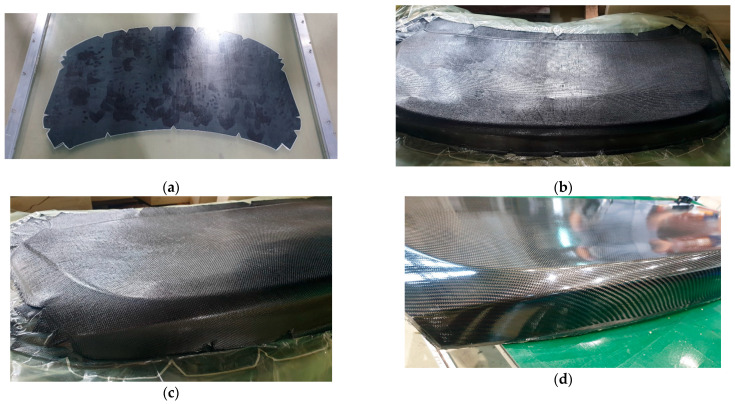
Product forming using mDDF: (**a**) mDDF frame; (**b**) preforming result; (**c**) preform result of high quality and (**d**) final product after PCM process.

After the diaphragm forming step, the shaped prepreg exhibited smooth surface contours and minimal evidence of out-of-plane deformation, as shown in Figure 14b. The use of the diaphragm films enabled more uniform pressure distribution, which allowed the prepreg layers to follow the mold curvature without severe fiber distortion. The trimmed preform was subsequently transferred to the PCM mold and subjected to full thermal curing under pressure to complete the molding process. The resulting molded part is presented in Figure 14c, which demonstrates the successful replication of complex geometry with high surface quality.

**Figure 15:**
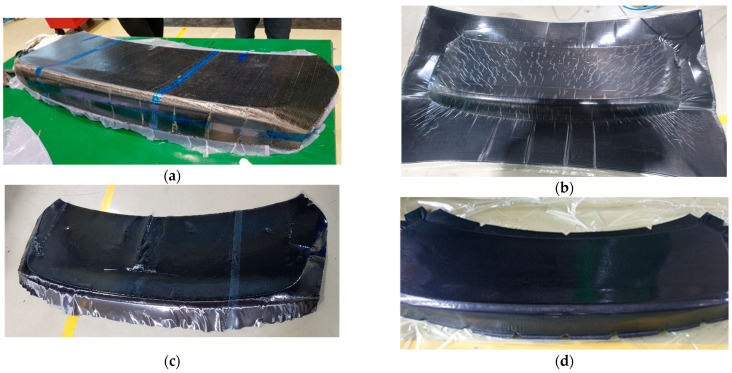
The results of preform product: (**a**) existing prepreg preforming; (**b**,**c**) before preforming product of wrinkle improvement using mDDF and (**d**) after preforming product of wrinkle improvement.

A comparison between molded components manufactured with and without the preforming stage is provided in Figure 15. Figure 15a shows the results of conventional preforming. Figure 15b shows the result of preforming the stacked prepreg using the Modified Double Diaphragm Forming (mDDF) method, while Figure 15c presents a more detailed view of the wrinkles that were not improved by this approach. In Figure 15d, a high-quality preform with reduced wrinkling is shown, achieved by applying a pre-cut stacked prepreg design optimized for wrinkle reduction.

These experimental results closely aligned [21] with the simulation predictions and confirmed the value of the mDDF approach in enhancing the formability of thermoset polymer matrix composites.

## 5. Discussion and Conclusion

### 5.1. Discussion

This study proposed and validated a Modified Double-Diaphragm Forming (mDDF) process to improve the forming quality of fiber-reinforced polymer composites fabricated via Prepreg Compression Molding (PCM). The approach integrates a semi-curing preforming stage at approximately 70 °C with diaphragm-assisted pressure application. This configuration enabled the forming of complex 3D geometries while maintaining fiber alignment and significantly minimizing wrinkle formation.

Simulations using AniForm Suite 3.0 were employed to predict wrinkle-prone regions and optimize the preform cut pattern. These simulations, based on material property data obtained through high-temperature mechanical characterization (Section 3), proved reliable. As shown in Figure 15a–d, wrinkle formation in 16 critical areas was predicted and experimentally confirmed prior to applying the mDDF process. After implementation, both simulation and preforming trials demonstrated that these wrinkles were completely eliminated.

**Figure 16:**
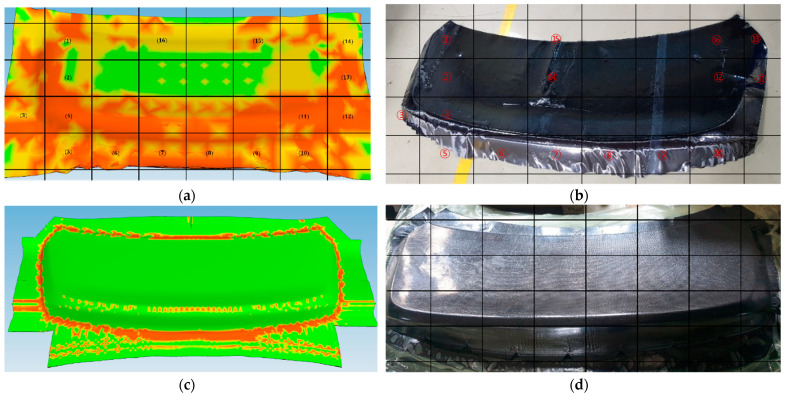
Correlation between simulation and experimental results: (**a**) predicted wrinkle formation (16) before mDDF preforming (simulation); (**b**) wrinkle defects (16) observed in the product before mDDF preforming (experiment); (**c**) predicted wrinkle reduction after mDDF preforming (simulation); (**d**) final product showing wrinkle elimination after mDDF preforming (experiment).

Furthermore, as illustrated in Figure 16, fiber alignment was visually consistent even across highly curved regions. While internal fiber orientation cannot be directly observed, it can be reasonably inferred from the external fiber layout and supported by draping analysis.

The effectiveness of mDDF depends on precise control of several key parameters, including the semi-cure temperature, preform geometry, and press timing. Additionally, the diaphragm material and vacuum bagging method played a crucial role in ensuring uniform pressure distribution. Future work should explore scaling the process to larger components and integrating automation for industrial application.

However, due to the absence of high-resolution surface quality inspection such as C-scan, it was not possible to perfectly evaluate all internal defects from the simulation and experimental results. Nevertheless, this study holds significance in that it addressed the key limitations and quality issues of the conventional PCM process (as shown in Figure 1b). Future work should include more advanced nondestructive inspection techniques to further validate and enhance the assessment of internal quality.

### 5.2. The Real-Life Application of Present Work

The mDDF process shows strong potential for practical use in the production of thermoset composite parts. By reducing wrinkle defects and maintaining fiber alignment, the process lowers defect rates and enhances mechanical properties, leading to material efficiency and increased productivity. However, the incorporation of a semi-curing stage and diaphragm forming step may increase overall processing costs.

Despite this challenge, the mDDF process is particularly well-suited for the aerospace industry, where manufacturing precision and structural quality take precedence over cost. With further optimization and cost reductions, the process may also be applied in the automotive industry, particularly for lightweight, high-strength structural components.

### 5.3. Conclusions

The Modified Double-Diaphragm Forming (mDDF) method presents an effective advancement over conventional PCM techniques for shaping thermoset fiber-reinforced polymer composites. By incorporating a controlled preforming step during the B-stage of epoxy curing, the process enables accurate shaping of complex geometries using pre-cut, stacked prepreg configurations designed to reduce wrinkling.

**Figure 17:**
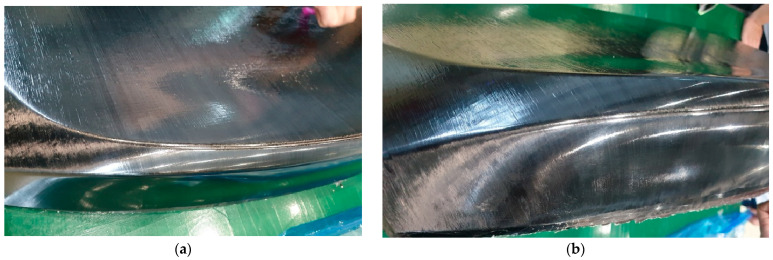
The results improvement of fiber orientation: (**a**,**b**) Fiber orientation observation after preforming.

Simulation and experimental validation confirmed that wrinkles—initially observed in 16 regions—were fully eliminated after mDDF application (Figure 15). The use of a semi-cured prepreg at ~70 °C ensured sufficient resin flow and shape stability, while uniform pressure contributed to wrinkle suppression. Fiber orientation (Figure 17) was preserved even on curved surfaces, as verified through visual inspection and draping analysis (Figure 16).

Mechanical testing and numerical analysis supported the robustness of the process, confirming its ability to improve forming quality, reduce material waste, and enhance dimensional accuracy. As such, mDDF contributes to the advancement of precision manufacturing in polymer matrix composites and expands the applicability of PCM to more complex, lightweight structures.

Although this research was conducted on an automotive component provided by an automobile manufacturer, the current cost structure limits near-term adoption in the automotive sector. Nonetheless, the mDDF process is expected to serve as a key enabling technology in the aerospace industry, where high structural quality is critical.

## Figures and Tables

**Table 1:** Bias extension test fitting data.

Bais Extension Test	Fitting Result	Iterations
Isotropic Elastic E (GPa)	0.46746	49
Newtonian Fluid η	0.20158	

**Table 2:** Friction test fitting data parameter results.

Friction Test	Fitting Result	Iterations
C	0.81611	1003
$\eta_{0}$	0.31804	
η	0.23889	
$a_{p}$	0.085598	
$b_{p}$	0.2509	
$p_{0}$	0.0033344	
$\tau_{0}$	0.0020944	

**Table 3:** Bending test data.

Bending Test	$0^{{}^{\circ}}$	$90^{{}^{\circ}}$
Overhang (o) Length (mm) avg.	0.0964	0.017
Areal weight (N/m^2^)	2.45	2.45
Thickness (mm)	0.27	0.27
$E_{b}$ (GPa)	0.17679	0.00097

**Table 4:** Key parameters by experimental values about preform.

Data	Experimental Values
Material Temperature (°C)	70
Pressure (MPa)	2
Upper mold weight (Kg)	79.7
Lower mold weight (Kg)	97.7

## Data Availability

The original contributions presented in this study are included in the article. Further inquiries can be directed at the corresponding author.

## References

[1] Chen S., Harper L. (2023). Two-dimensional to three-dimensional dry fibre preforming. Design and Manufacture of Structural Composites.

[2] Lee J.-M., Kim B.-M., Ko D.-C. (2019). Development of vacuum-assisted prepreg compression molding for production of automotive roof panels. Compos. Struct..

[3] Harper L.T., Turner T.A., Warrior N.A., Dahl J.S., Rudd C.D. (2006). Characterisation of random carbon fibre composites from a directed fibre preforming process: Analysis of microstructural parameters. Compos. Part A Appl. Sci. Manuf..

[4] Thornton M.J., Walker G.S. (2009). Catalytic carbon deposition on three-dimensional carbon fiber preforms using alkane gas feedstocks. New Carbon Mater..

[5] Yu F., Chen S., Harper L.T., Warrior N.A. (2021). Investigation into the effects of inter-ply sliding during double diaphragm forming for multi-layered biaxial non-crimp fabrics. Compos. Part A Appl. Sci. Manuf..

[6] Wang L., Wang J., Liu M., Peng X. (2020). Development and verification of a finite element model for double diaphragm preforming of unidirectional carbon fiber prepreg. Compos. Part A Appl. Sci. Manuf..

[7] Zhang H., Robitaille F., Grosse C.U., Ibarra-Castanedo C., Martins J.O., Sfarra S., Maldague X.P.V. (2018). Optical excitation thermography for twill/plain weaves and stitched fabric dry carbon fibre preform inspection. Compos. Part A Appl. Sci. Manuf..

[8] Wang Z., Wang B., Qin X., Dong S., Xu C., Meng S., Fang G. (2022). The effect of fiber preform configuration on in-plane compressive behavior of high porosity needled carbon/carbon composites in elevated temperature environment. Ceram. Int..

[9] Bersee H., Lindstedt S., Niño G., Beukers A. Diaphragm forming of thermoset composites. Proceedings of the 16th International Conference on Composite Materials, Kyoto International Conference Center.

[10] Zhang W., Bostanabad R., Liang B., Su X., Zeng D., Bessa M.A., Wang Y., Chen W., Cao J. (2019). A numerical Bayesian-calibrated characterization method for multiscale prepreg preforming simulations with tension-shear coupling. Compos. Sci. Technol..

[11] Dutta S., Körber M., Frommel C. (2019). Automated fixation of dry carbon fibre fabrics with RTM6 for autonomous draping and sensor-aided preforming. Procedia CIRP.

[12] Zhang W., Gao J., Cao J. (2020). Blank geometry design for carbon fiber reinforced plastic (CFRP) preforming using finite element analysis (FEA). Procedia Manuf..

[13] Li H. (1998). Forming of Advanced Thermoset Composites: Process Development and Deformation Study. Ph.D. Thesis.

[14] Alshahrani H., Hojjati M. (2016). Influence of double-diaphragm vacuum compaction on deformation during forming of composite prepregs. J. Sci. Adv. Mater. Devices.

[15] Pantelakis S.G., Baxevani E.A. (2002). Optimization of the diaphragm forming process with regard to product quality and cost. Compos. Part A Appl. Sci. Manuf..

[16] Gutowski T.G., Dillon G., Chey S., Li H. (1995). Laminate wrinkling scaling laws for ideal composites. Compos. Manuf..

[17] Facon E., Ivars J., Labanieh A.R., Salem M.M., Soulat D. (2024). Measurement device for tear defects during preforming of non-woven fabrics made of recycled carbon fibres. Compos. Part A Appl. Sci. Manuf..

[18] Wei K., Liang D., Mei M., Wang D., Yang X., Qu Z. (2019). Preforming behaviors of carbon fiber fabrics with different contents of binder and under various process parameters. Compos. Part B Eng..

[19] Belhaj M., Deleglise M., Comas-Cardona S., Demouveau H., Binetruy C., Duval C., Figueiredo P. (2013). Dry fiber automated placement of carbon fibrous preforms. Compos. Part B Eng..

[20] Chen S., McGregor O.P.L., Endruweit A., Elsmore M.T., De Focatiis D.S.A., Harper L.T., Warrior N.A. (2017). Double diaphragm forming simulation for complex composite structures. Compos. Part A Appl. Sci. Manuf..

[21] Potter K. (2002). Beyond the pin-jointed net: Maximising the deformability of aligned continuous fibre reinforcements. Compos. Part A Appl. Sci. Manuf..

